# Correlation between normal range of serum alanine aminotransferase level and metabolic syndrome: A community-based study

**DOI:** 10.1097/MD.0000000000012767

**Published:** 2018-10-12

**Authors:** Han Shen, Jing Lu, Ting-Ting Shi, Cheng Cheng, Jing-Yi Liu, Jian-Ping Feng, Jin-Kui Yang

**Affiliations:** a Department of Endocrinology, Beijing Tongren Hospital, Capital Medical University; b Beijing Key Laboratory of Diabetes Research and Care; c Beijing Diabetes Institute, Beijing, China.

**Keywords:** alanine transaminase, lipid metabolism disorders, metabolic syndrome

## Abstract

Serum alanine aminotransferase (ALT) is a biomarker of hepatocyte damage. However, the relationship between normal range of serum ALT level and metabolic syndrome (MetS) has not been completely understood. This study aimed to investigate the correlation between normal range of serum ALT level and MetS.

A total of 2453 participants from the Beijing Community Pre-Diabetes study were enrolled. Multiple linear regression analysis was performed to calculate the regression coefficient. Normal serum ALT levels were divided into quartiles. Logistic regression model was used to compare the relative risk of MetS, and the receiver operating characteristic (ROC) curve to calculate the optimal ALT boundary value for predicting MetS.

The frequency of MetS increased with the ALT level within the normal range. Compared with the first group, the risk of MetS was greater in the other quartiles of ALT level in males, the difference was significant for the fourth group. For females, the risk of MetS increased with ALT level within the normal range as well, with all differences showing statistical significance. The optimal ALT boundary value of the ROC curve for males and females was 24.5 and 14.5 U/L, respectively.

ALT was related to metabolic factors and used as one of the indicators to assess the morbidity risk of metabolic diseases.

## 1 Introduction

Metabolic syndrome (MetS) includes multifaceted metabolic disorders in the whole body, which is closely related to cardiovascular and cerebrovascular diseases as well as diabetes mellitus.^[1–10]^ The diagnostic factors include abdominal obesity and abnormal metabolic indexes such as blood pressure, blood glucose, blood lipid, and so on.^[11–15]^ Alanine aminotransferase (ALT) is a sensitive indicator of liver injury, which is commonly used to screen and detect the abnormal liver function and evaluate its abnormal degree.^[16]^ In China, the normal upper limit of ALT is usually 40 U/L. In a study on Jilin province, Hong et al found that the incidence of abnormal liver function was 17.53%; of which, nonalcoholic fatty liver disease accounted for 10.79% and MetS accounted for 16.25%, while patients suffering from both accounted for 20.31%. These 2 were the main causes of abnormal liver function.^[17]^ On the one hand, when patients suffer from MetS, excessive lipid is deposited in nonadipose tissue. Especially the infiltration in the liver damages the normal function of liver cells, resulting in the release and elevation of liver enzymes in blood. On the other hand, lipid deposition causes insulin resistance and the elevation of the levels of blood glucose, blood lipids, and so on, promoting changes in arteriosclerosis and increasing the incidence of cardiovascular events.^[18,19]^

Previous studies suggested a possible correlation between ALT and MetS, but the conclusions were not consistent. Some studies showed that the elevation of ALT level increased the morbidity risk of MetS, cardiovascular diseases, and diabetes.^[16,20]^ Moreover, a study also reported that, compared with the normal range of ALT level, the elevation of ALT was not a risk factor for the incidence of MetS.^[21]^ At the same time, some studies also indicated that the normal reference range of ALT was associated with MetS,^[22,23]^ but the correlation was different among different races and sexes.^[24–26]^ Therefore, the purpose of this study was to investigate whether the changes in the ALT level in the current normal reference range had a correlation with or predictive effect on metabolic diseases, including understanding the correlation between the normal ALT level and metabolic indexes (blood glucose, blood lipid, blood pressure, and so on). It also aimed to analyze the relationship between the normal ALT level and the incidence of MetS and to calculate the optimal boundary value of ALT.

## 2 Methods

### 2.1 Study population

All participants were from the Beijing Community Pre-Diabetes study in 2009. They were older than 18 years and belonged to the Nanfaxin Community of Beijing. Data, such as gender, age, height, weight, waist circumference (WC), blood pressure, blood lipid, fasting blood glucose (FPG), and serum ALT, were collected. The participants were asked to fill out a health questionnaire to understand their history of smoking, drinking, and other diseases. A total of 3722 participants were selected. The exclusion criteria were as follows: participants with positive surface antigen of the hepatitis B virus (HBsAg; n = 137), a history of drinking (n = 408), diabetes mellitus (n = 491) or incomplete data (n = 52), and with ALT >40 U/L (n = 181). At last, a total of 2453 participants were finalized, including 554 males and 1899 females.

This study was approved by the ethics committee of Beijing Tongren Hospital, Capital Medical University (Beijing, China). All participants signed the informed consent form.

### 2.2 Data collection

Indexes such as name, gender, age, and other general information of participants were recorded, and their blood pressure, weight (kilogram), and height (centimeter) were measured. Their weight was measured using a calibrated scale. The WC was measured at the midpoint between the highest point of iliac crest and the lowest part of costal margin in the midaxillary line. The body mass index (BMI) was the weight divided by the square of height. The blood sample of participants, who were fasted overnight for more than 8 hours, was collected. The ALT, HBsAg, FPG, and serum lipid profiles [including total cholesterol (TC), triglyceride (TG), low-density lipoprotein cholesterol (LDL-C), and high-density lipoprotein cholesterol (HDL-C)] were measured. All the participants were asked to fill out a questionnaire to understand their history of smoking, drinking, and other diseases.

### 2.3 Diagnostic criteria for metabolic syndrome

The definition of MetS referred to the definition of the National Cholesterol Education Program/Adult Treatment Panel III in 2005^[27]^ (with 3 or more than 3 items): central obesity (≥90 cm for males and ≥80 cm for females); TG ≥ 1.7 mmol/L; HDL-C decreased: <1.03 mmol/L for males and <1.30 mmol/L for females; blood pressure ≥130/85 mm Hg, or the corresponding treatment already received; and 5. FPG ≥ 5.6 mmol/L, or type 2 diabetes diagnosed and the corresponding treatment already received.^[28]^

### 2.4 Statistical analysis

SPSS17.0 (SPSS Inc., IL) was used for statistical analysis. All the participants were grouped according to gender. The differences in the indicators of the 2 groups were compared. The *t* test was used for continuous data, which were expressed as mean ± standard deviation (interquartile range). Multiple linear regression analysis was used to calculate the regression coefficient *β* and the 95% confidence interval (CI) of *β* to understand the correlation between normal ALT level and various metabolic indexes. Moreover, all the participants were grouped according to the quartiles of ALT value (U/L), and the first group was used as the reference group. The relative risk of MetS in each group was compared using the logistic regression model, age, and BMI were used for correction. The results were expressed in odds ratio (OR) value and 95% CI. The receiver operating characteristic (ROC) curve between MetS and ALT was plotted, and then the area under the curve was calculated for the optimal ALT boundary value to predict MetS. A *P* value <.05 was considered as significant difference.

## 3 Results

Around 554 males and 1899 females were enrolled, and the results indicated a difference between the serum ALT level of males and females. The serum ALT level of males was higher than that of females. The WC and diastolic pressure of males were greater than those of females, and HDL-C was lower than that of females (Table 1).

**Table 1:**
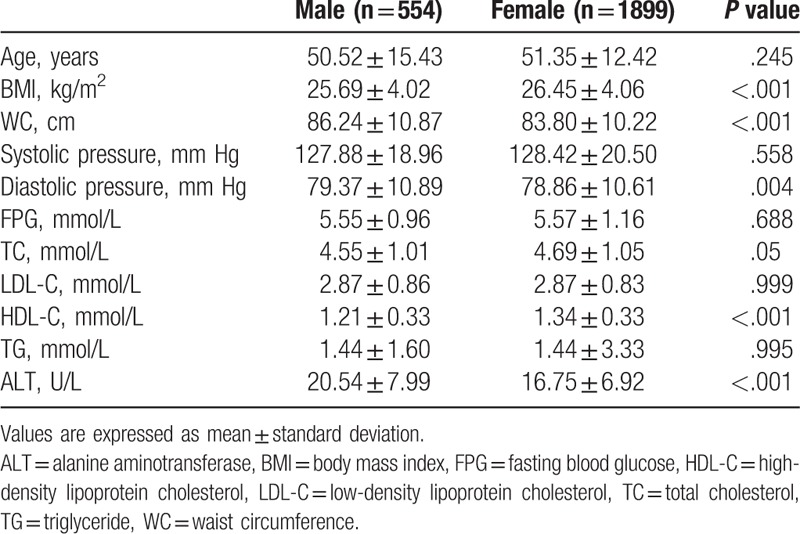
General characteristics of the study population.

The regression coefficient of the ALT level of males with WC, TC, and TG was 0.249, 0.83, and 0.523, respectively, which was significantly different. For females, a positive correlation was observed between the ALT level and BMI, WC, diastolic blood pressure, TC, and LDL-C, the results were significantly different. The regression coefficient of HDL-C was −2.043, which was also significantly different (Table 2).

**Table 2:**
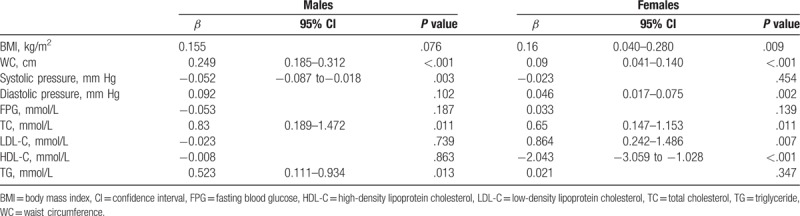
Results of multiple linear regression analyses of the relationship between metabolic factors and serum ALT level.

The participants were divided into 4 groups according to the quartiles of ALT value, and the first group was used as the reference group. The results showed that for males with ALT ≤40 U/L, the morbidity rate of MetS was 25.81%; of which, the morbidity rate in the first (1–14 U/L), second (15–19 U/L), third (20–25 U/L), and fourth groups (26–40 U/L) was 14.29%, 22.73%, 20.61%, and 45.14%, respectively. The morbidity rate of MetS increased with the increase in ALT. Further statistics showed that the first group was used as the reference group, and the OR value of the second, third, and fourth groups after age correction was 1.797, 1.732, and 6.702, respectively. Only the *P* value of the fourth group was always less than 0.05 before and after the correction. This association remained statistically significant after adjustment for age and BMI (OR value 6.388). Therefore, the morbidity rate of MetS for males in the fourth group was higher than that in the first group (Table 3).

**Table 3:**
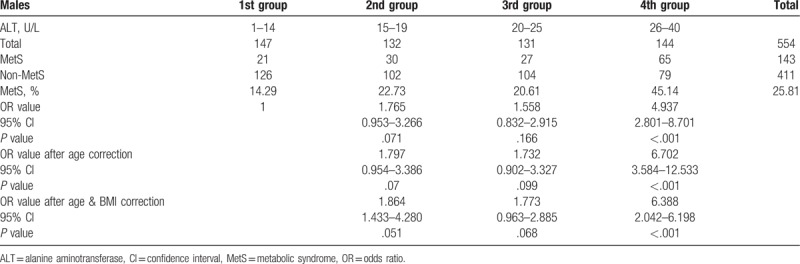
Logistic regression analyses of the relationship between metabolic syndrome presence and serum alanine aminotransferase level in males.

For females with ALT ≤40 U/L, the morbidity rate of MetS was 40.92%; the morbidity rate in the first (1–11 U/L), second (12–15 U/L), third (16–20 U/L), and fourth groups (21–40 U/L) was 23.09%, 35.63%, 47.56%, and 57.69%, respectively. Further statistics showed that the first group was used as the reference group, and the OR value of the second, third, and fourth groups after age correction was 1.787, 2.697, and 4.154, respectively, which was significantly different (*P < *.05). After adjusting for age and BMI, the association remained statistically significant. The OR values increased with the increase in ALT, indicating that the morbidity rate of MetS for females increased with the increase in ALT, further indicating a positive correlation between the 2, as shown in Table 4.

**Table 4:**
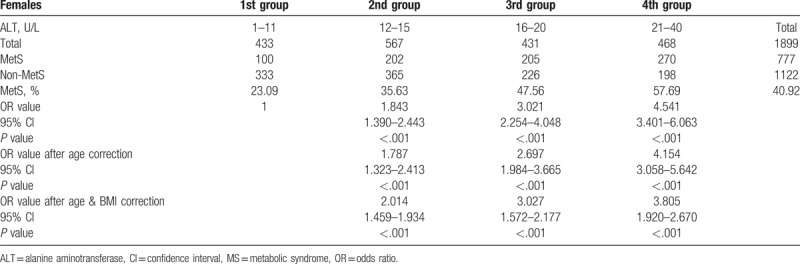
Logistic regression analyses of the relationship between metabolic syndrome presence and serum alanine aminotransferase level in females.

The ROC curve between MetS and ALT was plotted, and the area under the curve and the optimal ALT boundary value were calculated (Table 5). The area under the curve, optimal ALT boundary value, specificity, and sensitivity for males were 0.663 (*P < *.05), 24.5 U/L, 0.783, and 0.483, respectively. The area under the curve, optimal ALT boundary value, specificity, and sensitivity for females were 0.658 (*P < *.05), were 14.5 U/L, 0.563, and 0.687, respectively.

**Table 5:**
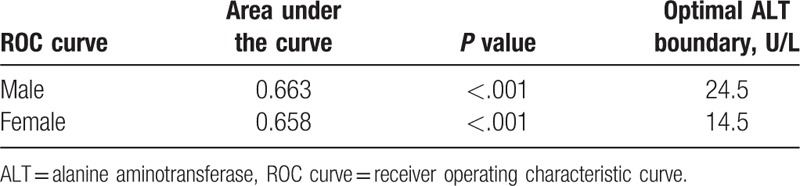
Thresholds for serum alanine aminotransferase values from receiver operating characteristic analyses.

## 4 Discussion

The present study found a positive correlation between the normal range of serum ALT level and the morbidity rate of MetS, which was still significant after age and BMI correction. The results were consistent with the results of some previous studies. The studies conducted in Israel and South Korea showed that in the normal reference range, the incidence of MetS increased with the increase in ALT.^[22,29]^ Another study conducted in South Korea indicated a significant correlation between ALT and MetS, and the correlation was still clear after insulin resistance and age correction.^[30]^ A study conducted in Japan found a strong correlation between elevated ALT and MetS. The elevated ALT threshold value of males and females was ≥30 U/L and ≥ 25U/L, respectively.^[31]^ A study conducted in the north of China showed that the ALT in the normal range or close to normal range was associated with metabolic disorders; the ALT boundary value for determining metabolic disorder was 21–25 U/L for males and 12 to 22 U/L for females. Moreover, a clear correlation existed between ALT and the morbidity rate of MetS.^[32]^ When ultrasound did not detect nonalcoholic fatty liver disease, MetS could cause a slight increase in ALT of diabetic patients. The WC and low HDL-C were related to transaminase. Moreover, oral hypoglycemic agents, antihypertensive drugs, and lipid-lowering drugs had little influence on this correlation.^[33]^

In the present study, males were divided into groups according to the quartiles of ALT, The first group was used as the reference group. After logistic regression, the OR value of the fourth group was 4.937, which after age and BMI correction was 6.388, indicating that at a normal high value of ALT level (i.e., the fourth group), the morbidity rate of MetS was relatively high. For females, the OR values in each group were statistically significant, and they increased with the increase in ALT. This further indicated that for the females in this study, a clear positive correlation existed between different ALT values in the normal range and the morbidity rate of MetS. With an increase in ALT, the morbidity rate of MetS increased gradually. Further, the ROC curve analysis showed that the optimal ALT boundary value for determining MetS was 24.5 U/L for males and 14.5U/L for females.

After finding a clear correlation between the morbidity rate of MetS and ALT in this study, the underlying reasons were analyzed. Jacobs et al^[34]^ suggested that insulin resistance was the main pathophysiological mechanism for the association of MetS with ALT, and the effects of adipose tissue-related inflammation and endothelial dysfunction were small. Aminotransferase is involved in transamination, and the concentration of glutamic acid and glutamine in the blood is closely related to insulin resistance, blood glucose, and MetS. The liver provides a place for the metabolism of amino acids.^[35]^ However, studies on the specific mechanisms of transaminase and MetS are scarce. In the present study, a large-scale investigation of the community in Beijing was conducted, although the correlation between ALT and blood glucose was not found. The potential significance of the normal reference range of ALT was preliminarily revealed in the MetS, that is, the predictive effect of MetS and the assessment of the morbidity risk of the disease. As far as we know, BMI and WC were simple and effective measures in predicting the risk of MetS. Serum ALT level was a conventional detection index in routine health check-ups in China. Our study revealed that mild elevations in serum ALT may be used to assess the morbidity risk of MetS to some extent. Consequently, serum ALT levels could be used as a supplementary indicator to predict the morbidity risk of metabolic diseases without increasing healthcare costs.

The present study had the following 3 limitations: Considering the effects of oral hypoglycemic drugs or insulin therapy on blood glucose, patients who had a history of diabetes were excluded, which might affect the study results of blood glucose. However, no correlation between blood glucose and ALT was found in this study. Therefore, more perfect and meticulous studies are needed in the future, such as recording the treatment of patients with diabetes, which might further reveal the relationship between blood glucose and liver enzymes. Insulin resistance might be an important link in relation to liver enzymes and metabolic disorders, but the evaluation and analysis of related data were not conducted in this study. The study had more female subjects than males. The participants in the study were from rural residents who lived in Nanfaxin Community for over 10 months every year. The shift of rural labor force to nonagricultural activities reduced the available male population. Therefore, more comprehensive consideration should be given in future study.

Serum ALT is not only a sensitive indicator of liver function but also closely related to metabolic factors. In this study, the community population was selected, and the relationship between multiple metabolic factors and the normal reference range of ALT, as well as the relationship between the morbidity rate of MetS and the normal reference range of ALT, was analyzed. The results indicated that in the normal reference range, the correlation between ALT and blood lipid was strong, and the morbidity rate of MetS gradually increased with the increase in ALT. Therefore, ALT could be used as one of the supplementary indicators to assess the morbidity risk of metabolic diseases.

## Author contributions

**Conceptualization:** Jin-Kui Yang.

**Data curation:** Han Shen, Jing Lu, Ting-Ting Shi, Cheng Cheng, Jing-Yi Liu, Jian-Ping Feng, Jin-Kui Yang.

**Funding acquisition:** Jin-Kui Yang.

**Investigation:** Jian-Ping Feng.

**Methodology:** Jin-Kui Yang.

**Project administration:** Jian-Ping Feng.

**Software:** Jing Lu.

**Supervision:** Jin-Kui Yang.

**Validation:** Ting-Ting Shi.

**Visualization:** Ting-Ting Shi.

**Writing – original draft:** Han Shen.

**Writing – review & editing:** Jing Lu.

Jinkui Yang orcid: 0000-0002-5430-2149.
